# Copper oxide–graphene oxide nanocomposite: efficient catalyst for hydrogenation of nitroaromatics in water

**DOI:** 10.1186/s40580-019-0176-3

**Published:** 2019-02-21

**Authors:** Kaiqiang Zhang, Jun Min Suh, Tae Hyung Lee, Joo Hwan Cha, Ji-Won Choi, Ho Won Jang, Rajender S. Varma, Mohammadreza Shokouhimehr

**Affiliations:** 1 Department of Materials Science and Engineering, Research Institute of Advanced Materials, Seoul National University, Seoul, 08826 Republic of Korea; 2 Center for Electronic Materials, Korea Institute of Science and Technology (KIST), Seoul, 02792 Republic of Korea; 3 Small & Medium Enterprises Support Center, Korea Institute of Science and Technology (KIST), Seoul, 02792 Republic of Korea; 4 Regional Centre of Advanced Technologies and Materials, Faculty of Science, Palacky University in Olomouc, Šlechtitelů 27, 783 71 Olomouc, Czech Republic

**Keywords:** Copper oxide, Graphene oxide, Hydrothermal, Hydrogenation, Synergistic effect

## Abstract

A low-cost nanocomposite catalyst containing copper oxide (CuO) nanoparticles (NPs) on graphene oxide (GO) was fabricated by a facile hydrothermal self-assembly process. The segregated CuO NPs and GO exhibited negligible catalytic activities for the reduction of nitroaromatics. However, their hybrid composite accomplished facile reduction with high conversions for several substituted nitroaromatics in aqueous NaBH_4_ solution; synergetic coupling effect of CuO NPs with GO in the nanocomposite catalyst provided excellent catalytic activity. The nanocomposite catalyst could be separated from the reaction mixture and recycled consecutively.

## Introduction

Catalysts play deterministic roles in the hydrogenation of nitroaromatics to aminoaromatics [1–3]. The development of highly active catalysts has attracted remarkable attention for the next-generation green, cost-effective and efficient reduction processes. In particular, the engineered metal nanoparticles (NPs) and nanocomposites have significantly improved the efficiency of catalytic systems. Consequently, various approaches have been utilized to fabricate the inexpensive nanostructured catalysts [4–7]. Varma et al. has emphasized the importance of the greener methods to synthesize nanocomposite catalysts [8]. Virkutyte et al. has reviewed various approaches for the fabrication of stable, environmentally benign, and active metal NPs for catalytic applications [9]. A summary of nanocatalysts utilized for the environmentally friendly reduction of nitroaromatics has also been reviewed by Zhang and colleagues, emphasizing the advantages of the introduced heterogeneous catalysts [10]. However, several drawbacks of the present catalysts need to be circumvented namely enhancing the specific surface area, long-term stability, production cost diminution, and ecological concerns pertaining to their industrial applications [11–14]. To fabricate such catalysts possessing the aforementioned competencies, various nanostructured catalysts have been designed and synthesized [15–17]; metal NPs have been stabilized on variable robust nanostructures, producing active nanocomposite catalysts for reduction of nitroaromatics [18–20]. These heterogeneous catalysts generally comprise precious metal nanocatalysts e.g. Pd, Pt, Rh, Ru etc. to achieve efficient hydrogenation of nitroaromatics [20–22]. However, they are not broadly utilized in the chemical industries due to their high costs which is critical issue from the economical viewpoint [23]. In comparison, earth abundant elements e.g. Cu, Co, Fe, Mn, and Ni are inexpensive and may serve as appropriate catalysts for several catalytic transformations, but with low catalytic activity [24–26]. The nano-sized counterparts of these catalysts also get aggregated quickly in the reaction media due to their high surface-to-volume ratio, limiting their efficiency and reusability [27, 28]. Consequently, engineering hybrid catalysts consist of nanocatalysts integrated susceptible supports is necessary to promote their activities. The nanocomposite catalysts often present improved catalytic properties by exhibiting synergetic effects between the supports and nanocatalysts [29–31].

Graphene has been extensively employed as a stable and excellent nanocatalysts support for synthesizing efficient heterogeneous catalysts [32, 33]; its exceptional conductivity can facilitate the electron transfer during the transformations [34]. Consequently, metal nanocatalysts supported on the graphene may potentially promote the reductants’ electrons donation in the reaction media enhancing the reduction efficiency. Indeed, the synergetic effect between less-reactive nanocatalysts and graphene leads to highly active hybrid nanocomposite catalysts [35].

Motivated by the aforementioned advantages, we synthesized an efficient nanocomposite catalyst consist of graphene oxide (GO) supported copper oxide NPs (CuO–GO) via a facile hydrothermal self-assembly process for the reduction of nitroaromatics. The CuO–GO nanocomposite catalyst exhibited high yields for the reduction of various nitroaromatics using aqueous sodium borohydride (NaBH_4_) at room temperature.

## Experimental

### Materials and characterization

Water was deionized by a Nano Pure System (Barnsted). The reagents used in this research were purchased from SigmaAldrich, Samchun, and Daejung and used without any further purification. X-ray photoelectron spectroscopy (XPS) was performed using an Al Kα source (Sigma probe, VG Scientifics) to characterize the surface chemical composition. The nanostructure of the prepared CuO–GO nanocomposite catalyst was studied using a high resolution X-ray diffraction (XRD, D8-Advance), a transmission electron microscope (TEM, JEOL JEM-3010) equipped with an energy-dispersive X-ray spectroscopy (EDX) detector, a scanning TEM (STEM, JEOL JEM-2100F), a thermal gravimetric analysis (TGA, simultaneous DTA/TGA analyzer), Raman technology (LabRAM HV Evolution), and a Fourier Transform-Infrared Radiation spectroscopy (FT-IR, Nicolet iS50). Gas chromatography-mass spectrometry (GC–MS, Agilent Technologies 7693 Autosampler and 5977A Mass selective detector) was employed to monitor the conversion ratio of the nitroaromatics to aminoaromatics.

### Preparation of GO

Graphene oxide was synthesized from graphite using the modified hummer’s approach [36]. Commercial graphite powder (10 g) was added into 230 mL concentrated H_2_SO_4_ and cooled to ~ 20 °C with a circulator. 300 g potassium permanganate was added while stirring. Then, the temperature of the reaction was adjusted to 40 °C and the mixture was stirred for 1 h. Water (500 mL) was added to the mixture and the temperature was increased to 100 °C. 2.5 mL H_2_O_2_ (30 wt%) was slowly added to the mixture. For purification, the suspension was washed with HCl solution (200 mL) using a filter and a funnel. The suspension was washed with water several times until the filtrate became neutral.

### Preparation of CuO–GO nanocomposite catalyst

CuO–GO nanocomposite catalyst was successfully synthesized using a facile hydrothermal self-assembly process by modifying a previously reported method [36]. CuCl_2_ (2 mmol) was dissolved in deionized water and mixed with the as-synthesized GO solution (15 mL) and transferred into a clean Teflon-lined container. Thereafter, the Teflon-lined container was filled (70% in volume) with deionized water, placed in an autoclave and tightly sealed, followed by heating up to 150 °C for 12 h. After gradually cooling down, the product was washed with sufficient deionized water and filtered for several times to remove the non- and/or poor-anchored copper oxide NPs on the GO.

### Catalytic reduction of nitroaromatics

The reduction of nitroaromatics to aminoaromatics was carried out using CuO–GO nanocomposite catalyst with aqueous NaBH_4_ as a reductant at room temperature. In a typical procedure, nanocomposite catalyst (50 mg) was dispersed in deionized H_2_O (30 mL). Then, a nitroaromatics (1 mmol), NaBH_4_ (1.2 mmol) and a small stirring bar were added into the reaction glass flask. The reaction mixture was stirred at room temperature for 30 min under air atmosphere. After completion of the reaction, the CuO–GO nanostructured catalyst was separated using a centrifuge. The yields of the aminoaromatics products were measured using a GC–MS. For the reusability evaluation of the nanocomposite catalyst, the separated catalyst was washed with deionized water and dried in an oven for the following runs. The cycling performance was achieved by repeating the above reduction process.

## Results and discussion

**Fig. 1:**
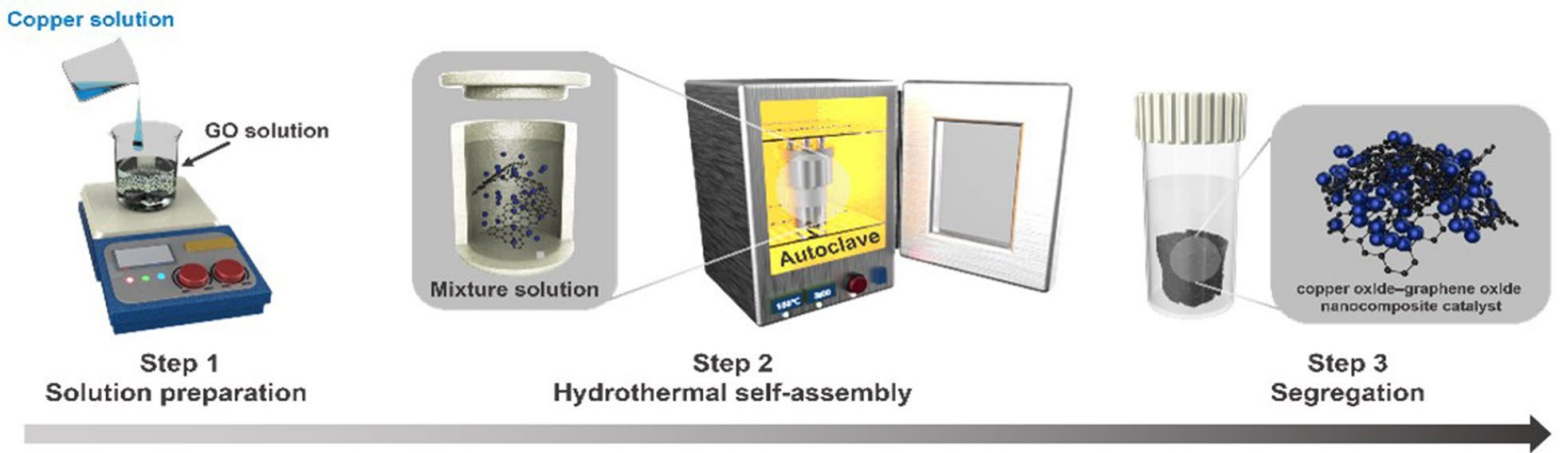
Schematic illustration for the synthesis of the CuO–GO nanocomposite catalyst

**Fig. 2:**
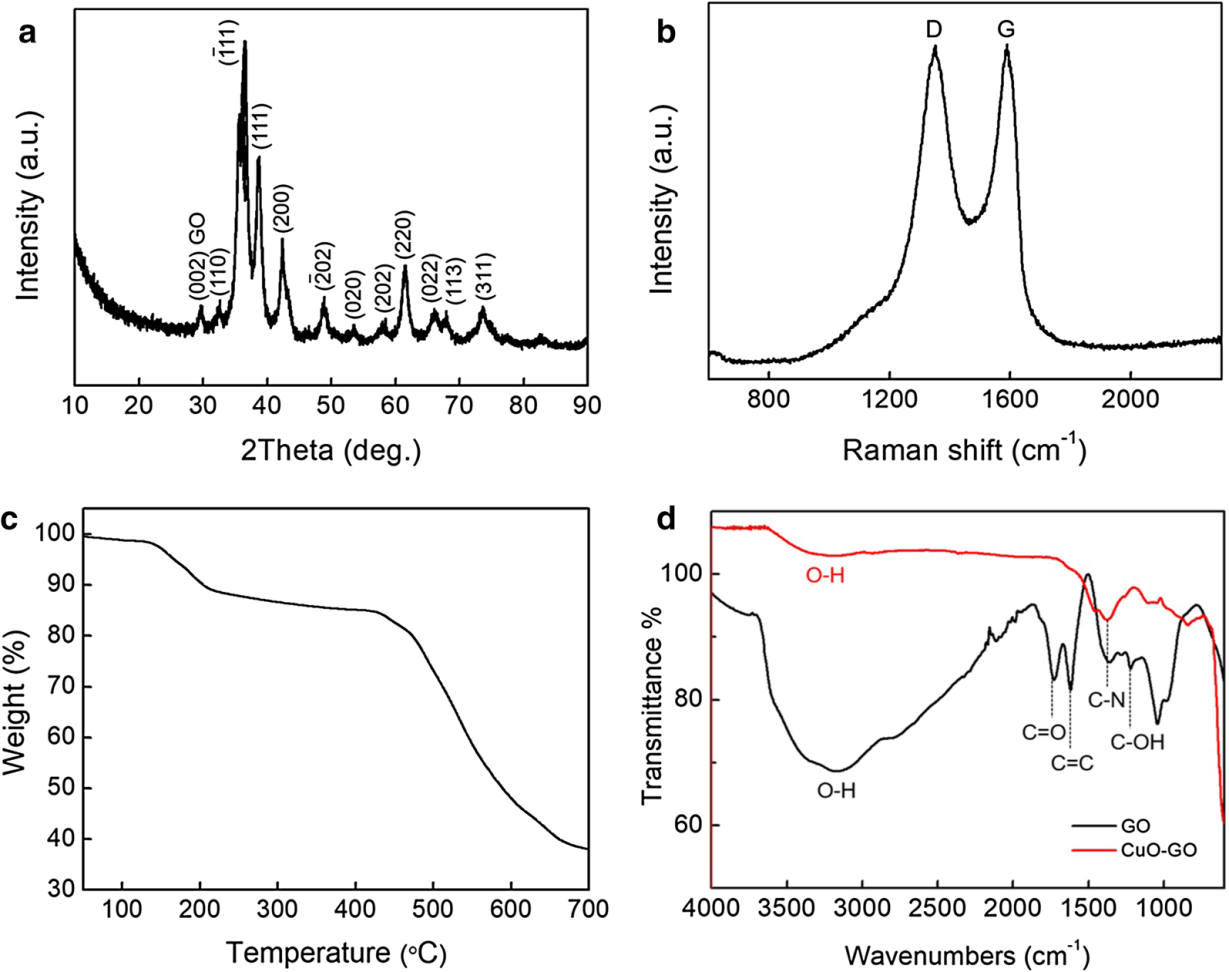
**a** XRD diffraction pattern, **b** Raman spectrum, **c** TGA analysis, and **d** FT-IR spectrum of the CuO–GO nanocomposite catalyst

Figure 1 depicts the overall synthetic procedure accomplished using a low-cost synthesis for CuO–GO nanocomposite catalyst. The XRD peaks (Fig. 2a) of the nanocomposite catalyst display are well indexed with GO and CuO (JCPDS Card no. 89-2530). The composition of the catalyst was further characterized by Raman technology to verify the GO support (Fig. 2b). The G line (first-order scattering of the E_2g_ phonons of sp^2^ orbital) at 1580 cm^−1^ and D line (ĸ-point phonons of A_1g_ symmetry) at 1350 cm^−1^ are clearly acquired for the nanocomposite catalyst as specific characteristics of graphene [37]. The thermal-stability of the catalyst was ascertained using TGA (Fig. 2c) under the nitrogen atmosphere with a temperature ramp of 10 °C min^−1^, demonstrating the presence of ~ 15 wt% moisture according to the first weight decrease and ~ 45 wt% graphene based on the second weight loss. Accordingly, weight percentage ratio of graphene to copper oxide species in the nanocomposite catalysts was found to be ~ 1:1.

The composition of the nanocomposite catalyst was further confirmed by FT-IR (Fig. 2d). A strong peak at 3450 cm^−1^ (O–H stretching vibrations) in the case of GO indicates H_2_O residual compared with CuO–GO nanocomposite catalyst even after sufficient drying. In addition, the characteristic bands of GO are clearly revealed at 1725 cm^−1^ for C=O stretching vibrations and 1600 cm^−1^ for C=C (skeletal vibrations of graphene). These peaks disappeared in the CuO–GO nanocomposite catalyst verifying the reduction of GO during the hydrothermal process.

**Fig. 3:**
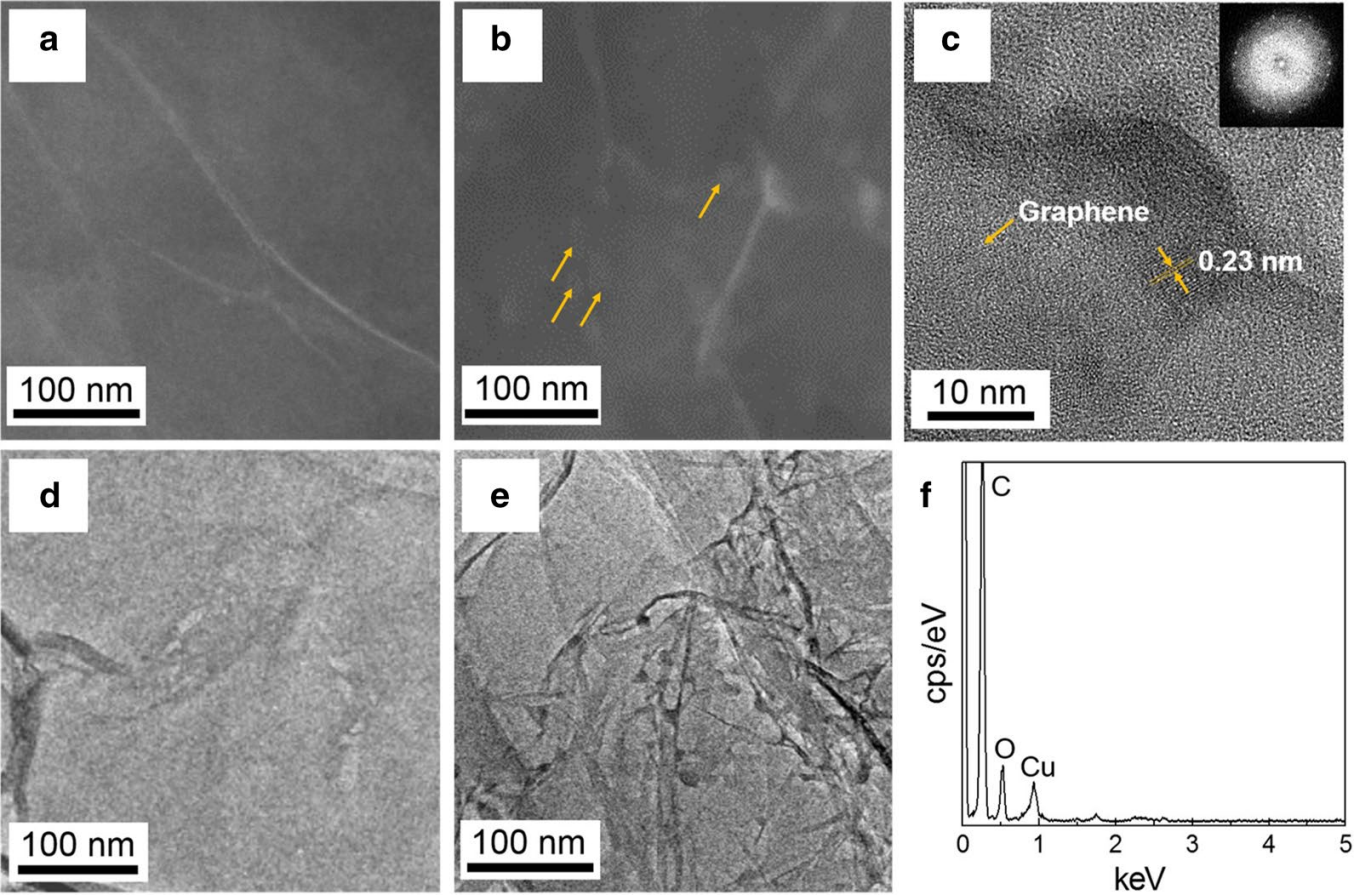
**a**, **d** TEM and STEM images of GO. **b**, **e** TEM and STEM images of the CuO–GO nanocomposite catalyst. **c** HRTEM image of the CuO–GO nanocomposite catalyst. The inset shows the relevant FFT image. **f** EDX spectrum of the CuO–GO nanocomposite catalyst

The STEM and TEM images (Fig. 3b, e) of the CuO–GO heterogeneous nanocomposite catalyst clearly revealed a uniform distribution of copper oxide NPs in comparison with GO sheets (Fig. 3a, d) after the hydrothermal self-assembly process. The high resolution TEM and fast Fourier transform (FFT) images (Fig. 3c) of the nanocomposite catalyst further provided valuable evidences for the spatial homogeneously confined and crystalline copper oxide NPs immobilized on GO framework. The crystalline NPs with the size of ~ 10 nm in diameter present nano-scale CuO grains with interplanar spacing of 0.23 nm on GO. The small size NPs expose high active sites on the surface, facilitating highly efficient hydrogenation. The EDX spectrum results determined the existence of Cu and O elements distribution on GO support (Fig. 3f).

**Fig. 4:**
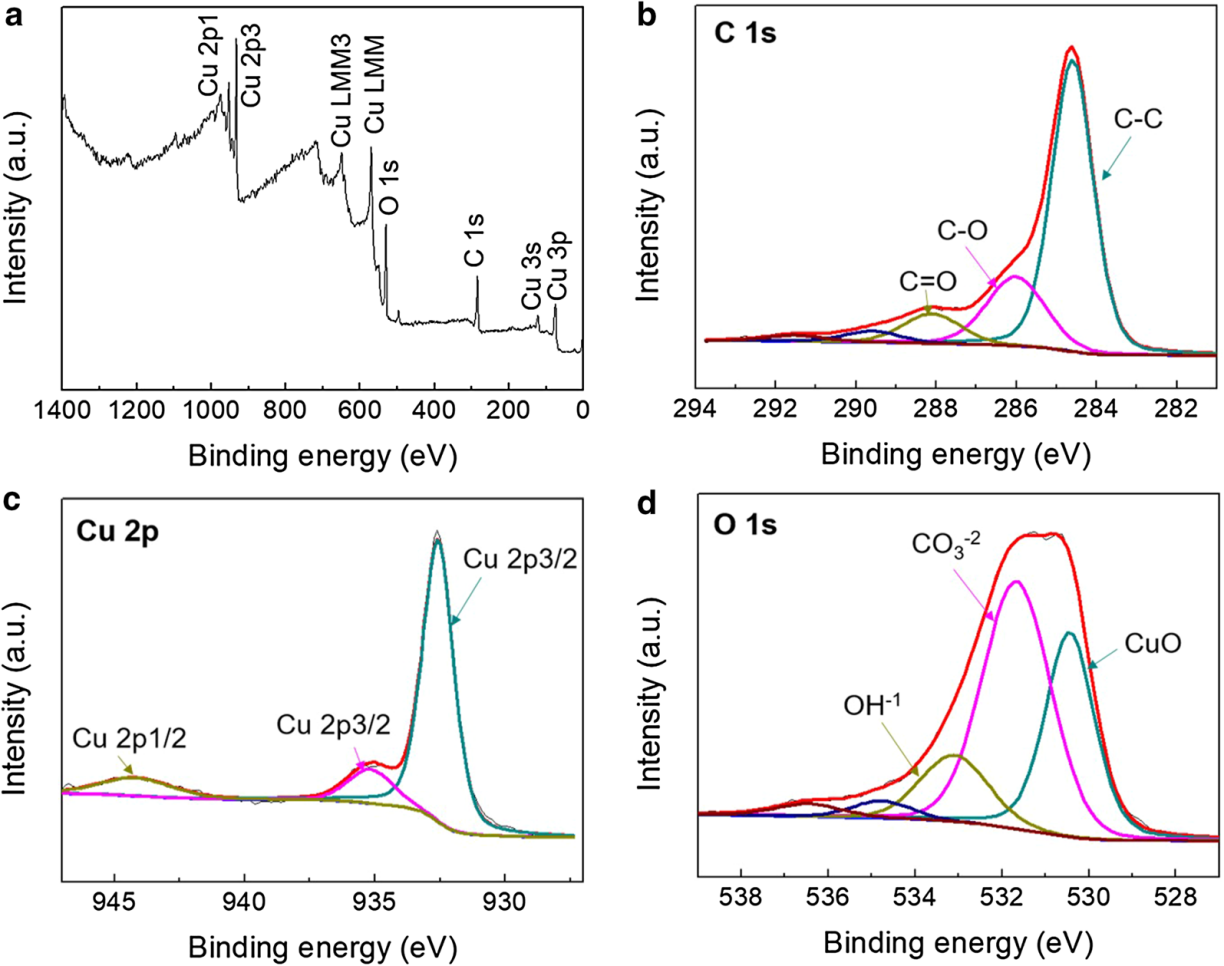
XPS analysis **a** survey scan, **b** C 1s, **c** Cu 2p, and **d** O 1s for the CuO–GO nanocomposite catalyst

The surface composition and existing elements on the nanocomposite catalyst were confirmed by XPS (Fig. 4). The survey spectrum (Fig. 4a) shows the surface binding situation, ascertaining the presence of Cu, O, and C elements in the nanocomposite catalyst (Fig. 4b–d). Carbon peaks are deconvoluted into three peaks, where the primary C–C bond generated by sp^2^ orbital hybridization shown at 284.6 eV. The oxide graphene is further verified by the C–O bond at 286.2 eV. Cu has binding energy of 944.3 eV (Cu 2p_1/2_) and 935.8, 932.8 eV (Cu 2p_3/2_) with O 1 s possessing binding energy of 530.6 eV thus demonstrating the existence of copper oxide in the nanocomposite catalyst. The other two peaks with binding energies of 533.2 eV and 531.8 eV can be ascribed to the absorption of water and oxygen molecules from environment. Others deconvoluted peaks with much lower intensities (536.6 eV and 535.8 eV) are the satellite peaks of C.

**Fig. 5:**
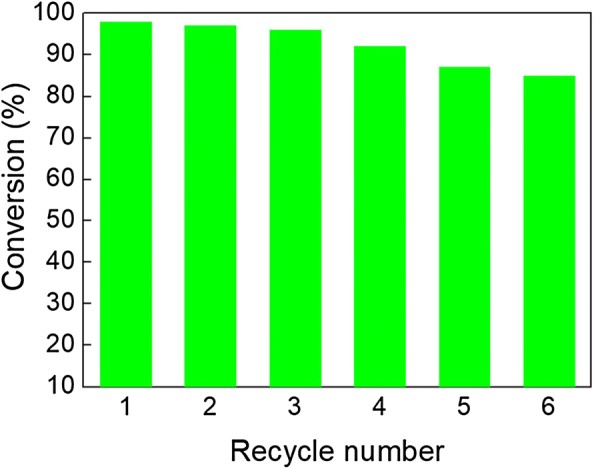
Reuse of the CuO–GO nanocomposite catalyst in the heterogeneous reduction of 4-nitrotoluene. Reaction conditions: 4-nitrotoluene (1 mmol), NaBH_4_ (1.2 mmol), catalyst (50 mg), room temperature, aqueous solution, and 30 min. The yields were determined by GC–MS

The catalytic activity of the nanocomposite catalyst was first examined in the reduction of 4-nitrobenzene with aqueous NaBH_4_ as a model reaction; high yield of conversion of 4-aminobenzene (98%, Table 1) was achieved. Although the selective reduction of a nitro moiety in the nitroaromatic compounds comprising another reducible functional group is a difficult transformation, nanocomposite catalyst selectively reduced the nitro to the amino moiety (Table 1, entries 2–7), demonstrating the feasibility of the synthesized catalyst for broad catalytic reduction applications. The excellent catalytic activity of the catalyst can be ascribed by the synergetic effect between copper oxide NPs and graphene oxide [38]. To highlight the catalytic activity of the CuO–GO nanocomposite catalyst in reduction of nitroaromatics, we compared the previously reported articles (Table 2) with the current catalyst. The CuO–GO nanocomposite catalyst showed less catalytic activity in comparison with other heterogeneous catalysts having noble-metals e.g. Pd, Pt etc. However, considering the low price of copper, a competitive catalytic activity was exhibited by the CuO–GO nanocomposite catalyst. Furthermore, the reusability of heterogeneous catalysts is necessary for its pragmatic usages [39–45]. The CuO–GO nanocomposite catalyst was successfully reused for six consecutive cycles of the reduction of 4-nitrotoluene with a good yield of 85% (Fig. 5).

**Table 1:**
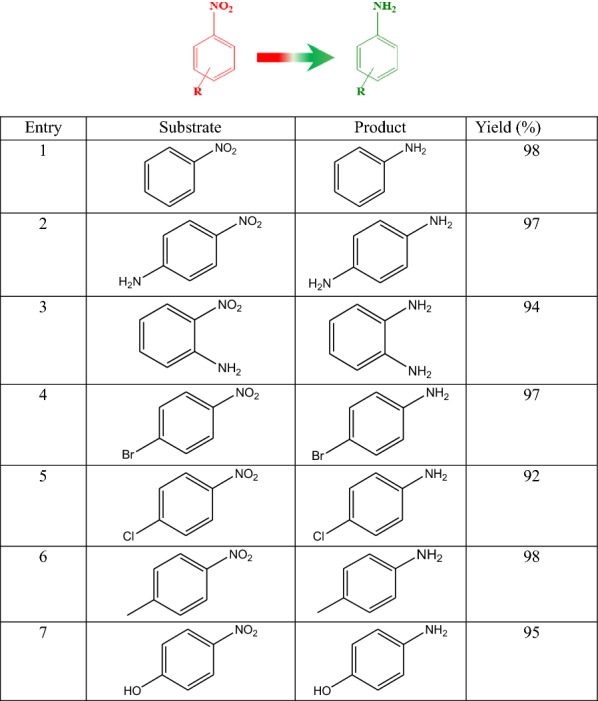
Heterogeneous reduction of substituted nitroaromatics catalyzed by CuO–GO nanocomposite catalyst in aqueous solution

Reaction conditions: nitroaromatics (1 mmol), NaBH_4_ (1.2 mmol), 30 min, CuO–GO nanocomposite catalyst (50 mg), aqueous solution. The yields were determined by GC–MS

**Table 2:** Catalytic comparison study of known heterogeneous catalysts in the reduction of nitrophenol

Entry	Catalyst	Reductant	Time (min)
1	Graphene–Cu_36_Ni_64_	NH_3_BH_3_ (3 mmol)	30 [37]
2	Cu–Ni–AAPTMS@GO	NaBH_4_ (1.5 mmol)	4 [38]
3	Ru_50_Ni_50_/RGONCs	NH_3_BH_3_ (2 mmol)	4.5 [39]
4	Pt/RGO	H_2_ (1 MPa)	120 [40]
5	(Co_6_)Ag_0.1_Pd_0.9_/RGO	HCOONH_4_ (4 mmol)	20 [41]
6	Pd/GO	NaBH_4_ (1.2 mmol)	10 [42]
7	MRN–Pd	NaBH_4_ (1.2 mmol)	45 [43]
8	CuSO_4_/GO	NaBH_4_ (1.2 mmol)	30 [This work]

## Conclusions

Small copper oxide NPs (~ 10 nm) formed nanocomposite with graphene oxide by a facile and cost-efficient hydrothermal self-assembly approach. Although GO and CuO NPs separately showed low catalytic activities in the reduction of nitroaromatics, their composite presented excellent reduction performance with high yield and selectivity for the conversion of various nitroaromatics bearing different functional groups, which can be described by the synergetic effect. In addition, the nanocomposite catalyst could be recycled for up to six uses. This system can be a promising heterogeneous catalyst for the future reduction of nitroaromatics premeditated in large scale wherein the low-cost and facile fabrication are demanded.
